# Differential effects of single fatty acids and fatty acid mixtures on the phosphoinositide 3-kinase/Akt/eNOS pathway in endothelial cells

**DOI:** 10.1007/s00394-022-02821-2

**Published:** 2022-02-14

**Authors:** Kim G. Jackson, Katie J. Newens, Michael J. Fry, Abby K. Thompson, Christine M. Williams

**Affiliations:** 1 Hugh Sinclair Unit of Human Nutrition, Department of Food and Nutritional Sciences, University of Reading, Harry Nursten Building, Reading, RG6 6DZ UK; 2 Institute for Cardiovascular and Metabolic Research, University of Reading, Reading, RG6 6AS UK; 3 School of Biological Sciences, University of Reading, Hopkins Building, Reading, RG6 6UB UK

**Keywords:** Aortic endothelial cells, eNOS phosphorylation, Dietary fatty acids, Non-esterified fatty acids, Oleic acid, Real time RT-PCR

## Abstract

**Scope:**

Dietary fat composition is an important modulator of vascular function. Non-esterified fatty acids (NEFA) enriched in saturated fatty acids (SFA) are thought to reduce vascular reactivity by attenuating insulin signalling via vasodilator pathways (phosphoinositide 3-kinase (PI3K)/Akt/endothelial nitric oxide synthase (eNOS)) and enhancing signalling via pro-inflammatory pathways.

**Methods:**

To examine the effects of fatty acids on these pathways, human aortic endothelial cells were incubated with single fatty acids, and mixtures of these fatty acids to mimic typical NEFA composition and concentrations achieved in our previous human study. RNA was extracted to determine gene expression using real-time RT-PCR and cell lysates prepared to assess protein phosphorylation by Western blotting.

**Results:**

Oleic acid (OA, 100 µM) was shown to down regulate expression of the insulin receptor, PTEN and a PI3K catalytic (p110β) and regulatory (p85α) subunit compared to palmitic, linoleic and stearic acids (*P* < 0.04), and promote greater eNOS phosphorylation at Ser^1177^. Both concentration and composition of the SFA and SFA plus n-3 polyunsaturated fatty acids (PUFA) mixtures had significant effects on genes involved in the PI3K/Akt pathway. Greater up-regulation was found with 800 than 400 µM concentration (respective of concentrations in insulin resistant and normal individuals), whereas greater down-regulation was evident with SFA plus n-3 PUFA than SFA mixture alone.

**Conclusion:**

Our findings provide novel insights into the modulation of the PI3K/Akt/eNOS pathway by single fatty acids and fatty acid mixtures. In particular, OA appears to promote signalling via this pathway, with further work required to determine the primary molecular site(s) of action.

**Supplementary Information:**

The online version contains supplementary material available at 10.1007/s00394-022-02821-2.

## Introduction

Elevated levels of non-esterified fatty acids (NEFA), characteristic of insulin resistant states such as obesity and type II diabetes, have been proposed to mediate their effects on vascular function by modulating insulin signalling pathways in the endothelium. In support of this, Lind et al. [1] reported the impairment in forearm blood flow during NEFA elevation to be reversed by insulin infusion in healthy subjects. In a study performed in rats, biopsies taken from the aorta showed evidence of NEFA-induced impairment of the phosphoinositide 3-kinase (PI3K) pathway, with a significant reduction in the phosphorylation of insulin receptor substrate-1 (IRS-1), Akt and endothelial nitric oxide synthase (eNOS), together with impaired responses to the endothelial-dependent vasodilator, acetylcholine [2]. Both studies achieved NEFA elevation using a commercially available triacylglycerol infusion, Intralipid, which is rich in n-6 polyunsaturated fatty acids (PUFA). Our previous human study found the experimental elevation of NEFA after feeding a test oil containing saturated fatty acids (SFA) with long chain (LC) n-3 PUFA to reverse the impairment in flow-mediated dilatation observed with SFA alone [3], confirming previous suggestions that dietary fat composition may be an important modulator of vascular function. Studies in endothelial cells examining the impact of dietary fat composition have reported more detrimental effects of SFA (palmitic acid (PA)) than MUFA (oleic acid (OA)) and n-6 PUFA (linoleic acid (LA)) on the phosphorylation status of the PI3K pathway and nitric oxide production [4–7] following insulin stimulation. In visceral adipocytes, OA has been shown to protect against insulin resistance by regulating the expression of genes in the PI3K signalling pathway [8], suggesting that a similar mechanism may also operate in the endothelium.

The mitogen-activated protein kinase (MAPK) pathway, which leads to the enhanced expression of the vasoconstrictor endothelin-1 and cell adhesion molecules, has also been shown to be activated following insulin binding to endothelial cells. Although dietary fat composition has been reported to have an impact on inflammatory gene expression in endothelial cells [7, 9–12], there is considerable disparity in the findings in the literature. A study performed by our group using human umbilical vein endothelial cells has shown PA, OA and LA to up-regulate cell adhesion molecule expression, whereas docosahexaenoic acid (DHA) tended to down-regulate expression [11]. In vitro studies have indicated that fatty acids may mediate some of their effects on cell adhesion molecule expression by activating inhibitor-κβ kinase-β (IKKβ), a serine kinase that regulates the pro-inflammatory transcription factor nuclear factor kappa-B (NFκB) [4, 12], but which also uses IRS-1 as a substrate for serine phosphorylation [13, 14]. Fatty acids were shown to induce IKKβ activity in a dose dependent manner, with PA showing greater activation than OA and LA, attenuating insulin-mediated eNOS phosphorylation and reducing nitric oxide production [4]. IKKβ may therefore provide a common molecular locus through which fatty acids mediate their effects on inflammation and vascular function.

Many of cell studies conducted to date have been performed using a select range of fatty acids which have been tested individually. This limits their extrapolation to the normal physiological state wherein the composition of NEFA comprises a mixture of fatty acids reflecting diet and adipose tissues compositions. The present in vitro study of human aortic endothelial cells (HAEC) had two aims; (1) to determine the impact of single fatty acids on the gene expression of key proteins involved in insulin signalling in endothelial cells, and, (2) to use mixtures of fatty acids which mimic plasma NEFA compositions and concentrations achieved in our human study (Clinicaltrials.gov NCT01351324) which compared acute effects of test oils rich in SFA and SFA with LC n-3 PUFA on vascular reactivity [3]. The findings provide novel insights into the regulation of the PI3K dependent pathway by OA in endothelial cells, with down-regulation of the gene expression of the PI3K subunits (p110β and p85α) and phosphatase and tensin homolog (PTEN) associated with greater eNOS phosphorylation at Ser^1177^. In addition, our findings also contribute to our understanding how the concentration and composition of fatty acid mixtures modulate the PI3K/Akt pathway in the endothelium.

## Materials and methods

### Cell culture

Clonetics™ single donor HAEC (Lot no. 5F1352.1, Lonza Biologics Plc, Slough, UK) were maintained in complete medium; endothelial basal medium-2 with SingleQuot™ supplement and growth factors [hydrocortisone, human fibroblast growth factor-B, vascular endothelial growth factor, insulin-like growth factor-1, human epidermal growth factor, gentamicin sulphate amphotericin B-1000, heparin, ascorbic acid and 2% (v/v) foetal bovine serum (FBS)]. Only endothelial cells passaged less than four times were used for experiments.

To examine the acute and chronic effects of single fatty acids on the mRNA expression of genes involved in the insulin signalling pathway and vascular function, endothelial cells were incubated for 3 and 24 h in the presence of the fatty acid-BSA complexes. Fatty acid mixtures were only incubated with cells for 3 h to mimic the design of our human study. For the 3 h (acute) incubations, cells were grown to 80% confluence in six-well plates and then incubated in large vessel endothelial cell basal medium (LVM; TCS CellWorks, Buckingham, UK) containing 2% (v/v) FBS and minimal growth factor supplements (heparin, hydrocortisone, human epidermal growth factor and fibroblast growth factor) for 21 h. The cells were then washed with HEPES buffered saline solution before incubation with serum-free LVM containing the fatty acid treatments for 3 h. For the 24 h (chronic) incubations, the treatments were included in both the 21 h incubation with LVM containing 2% (v/v) FBS and the 3 h incubation with serum-free LVM. Since these cell experiments were also used to determine the effects of the fatty acid incubations on the phosphorylation status of selected proteins in the insulin signalling pathways, cells were incubated with Krebs–Ringer-HEPES buffer [15] for 1 h before RNA extraction or insulin stimulation.

### Fatty acid treatments

Sodium salts of PA, OA, LA, SA, eicosapentaenoic acid (EPA) and DHA (Sigma) were used to prepare stock solutions of the fatty acid-BSA complexes (molar ratio of 2.5:1) as previously described [11]. The fatty acid-BSA stock solutions were filter sterilised before the concentration of the fatty acids within the stock solutions were verified following extraction of the lipids and separation of the fatty acid methyl esters using gas chromatography [16]. The fatty acid-BSA stock solutions were then stored in aliquots at − 20 °C until dilution to the appropriate concentration in the LVM. Cells were treated with physiological concentrations (100 µM) of the single fatty acids PA, OA, LA and SA, with a lower concentration (10 µM) for the LC n-3 PUFA, EPA and DHA. Mixtures of fatty acid representative of the peak postprandial plasma NEFA profiles reached during the acute human study [3] were prepared for: (1) the SFA challenge (% weight of total fatty acids, 39% PA, 10.7% SA, 36.8% OA, 12.0% LA, 0.2% EPA and 1.2% DHA) and (2) the SFA with LC n-3 PUFA challenge (37.1% PA, 10.1% SA, 33.9% OA, 11.7% LA, 0.9% EPA and 6.2% DHA). The total NEFA concentrations chosen for study were 400 µM and 800 µM to reflect the circulating postprandial NEFA concentrations reported in healthy and insulin-resistant individuals, respectively [17, 18].

### Real time RT-PCR

Total RNA was extracted from the cells using Qiagen RNeasy Mini Kit and QIAshredder (Qiagen Ltd., Crawley, UK) using protocols recommended by the manufacturer. cDNA was generated from 0.5 to 1.5 μg samples of total RNA at 42 °C for 50 min (reaction volume 20 μl) using oligo dT_12–18_ primer (Invitrogen Ltd, Paisley, UK) and reverse transcriptase (Superscript II; Invitrogen Ltd) using protocols recommended by the manufacturer.

Specific primers for PTEN, IKKβ, Akt2, PI3K catalytic (p110α and β) and regulatory (p85α and β) subunits were designed (Table 1) and real time RT-PCR performed as previously described [19]. The primer sequences for eNOS was obtained from Miyomoto et al. [20] and vascular cell adhesion molecule-1 (VCAM-1) from Shaw et al. [11]. The expression of each target gene was normalized to the β-actin expression and the data represent the fold change in mRNA expression relative to the BSA control (HAEC incubated with BSA equivalent to the amount present in the fatty acid-BSA complexes), which is arbitrarily defined as 1.

**Table 1:** Primer sequences of genes for the relative quantification of mRNAs by real-time RT-PCR

Gene	Accession number	Sequence of the forward and reverse primers (5′–3′)	Product size (bp)
Akt2	BC120995	CATGAAGATCCTGCGGAAGG GAAGGCATACTTCAGCGCAGTG	121
IKKβ	AF031416	GCTTGAAGGCCAGAATCCAAC GGCCCTCATTTAACTTGCC	136
IR	M10051	CCAGTGATGTGTTTCCATGCTC GCCTCACCCTTGATGATGTC	146
p85α	BC094795	GATTGTGATACACCCTCCGTG GCTGGAATGACAGGATTTGG	104
p85β	BC070082	GCAGGACCAGATTGTCAAGG GCCTCAATTGCAGTACGCTTC	163
p110α	BC113601	GTACCTTGTTCCAATCCCAGG GTTCCTCTTTAGCACCCTTTCG	131
p110β	BC114432	GCTGGTTTGGATCTTCGGATG CTTTGTTGAAGGCTGCTGCAG	148
PTEN	BC005821	GTTCAGTGGCGGAACTTG GAACTTGTCTTCCCGTCGTG	101

*Akt2* v-akt murine thymoma viral oncogene homologue 2, *IKKβ* inhibitor-κβ kinase-β, *IR* insulin receptor, PI3K regulatory (p85α and p85β) and catalytic (p110α and p110β) subunits, *PTEN* phosphatase and tensin homolog

### Cell studies to determine the effects of the fatty acid treatments on insulin signalling

To determine whether changes in gene expression could have an impact on the phosphorylation status of key proteins in the PI3K (eNOS Ser^1177^ and Akt^473^) and MAPK (p44/p42 at Thr^202^/Thr^204^) pathways, cells were also stimulated with 1 µM of insulin (Novo Nordisk, Denmark) or HBSS for 15 min after incubation with the single fatty acids (PA, OA, LA, SA, EPA and DHA) and fatty acid mixtures (SFA and SFA with LC n-3 PUFA). This concentration of insulin was chosen based on a previous study determining insulin signalling in HAEC [15]. Cells were then placed on ice and incubated with 200 µl of 1X lysis buffer (20 mM Tris–HCl (pH 7.5), 150 mM NaCl, 1 mM Na_2_EDTA, 1 mM EGTA, 1% Triton, 2.5 mM sodium pyrophosphate, 1 mM *β*-glycerophosphate, 1 mM Na_3_VO_4_ and 1 µg/ml leupeptin) (New England Biolabs, Hitchin, UK) supplemented with 1 mM PMSF for 5 min before being scraped from the dishes.

Immunoblotting was performed as previously described [21]. Briefly, samples (30 µg protein) were run on 7.5% SDS–polyacrylamide gels and proteins transferred to nitrocellulose membranes (Hybond-ECL; Amersham, Buckinghamshire, UK) by semi-dry electroblotting (1.5 mA/cm^2^). Blots were incubated with primary antibodies for anti-phospho-eNOS (Ser^1177^, 1:1000 dilution), anti-phospho-Akt (Ser^473^, 1:1000) or anti-phospho-MAPK 44/42 (Thr^202^/Thr^204^, 1:10,000) and anti-eNOS (1:1000), anti-Akt (1:1000) or anti-MAPK 42/44 (1:10,000 dilution)(New England Biolabs) in Tris-Tween Buffered Saline (TTBS) containing 5% (w/v) BSA overnight at 4ºC on a three-dimensional rocking table. Proteins were detected using a 1:1000 dilution of anti-rabbit IgG conjugated to horseradish peroxidise (Sigma) in TTBS containing 1% (w/v) skimmed milk powder for 45 min and then exposed to ECL-reagent for 1–2 min and developed. The molecular weight of the bands was estimated by comparison with pre-stained molecular weight markers (MW 10–250 kDa, Bio-Rad, Hemel Hempstead, UK) that were run in parallel with the samples. Bands were imaged using Image J software (National Institute of Health, Maryland, USA) and the results calculated as the ratio of phosphorylated to total protein relative to the BSA control, which is arbitrarily defined as 100%.

### Statistical analysis

Data were analysed using IBM SPSS statistics version 25. The fold changes in gene expression and ratios of phosphorylated to total protein were analysed using a mixed-factor ANOVA and results are presented in the text and figures as mean ± SEM. The data were checked for normality and log-transformed where necessary to render their distribution normal before statistical analysis. When statistical differences were found in the ANOVA, a LSD posthoc test was applied to identify differences amongst the fatty acid treatments. Values of *P* ≤ 0.05 were taken as significant.

## Results

### OA has significant effects on the PI3K/Akt/eNOS pathway

**Fig. 1:**
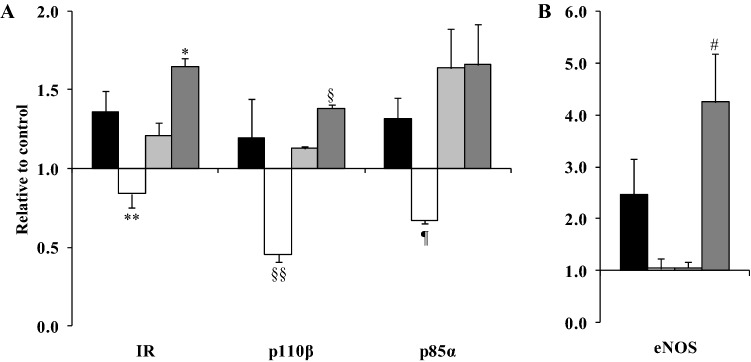
**A** OA down-regulates the expression of the insulin receptor (IR), regulatory (p110β) and catalytic (p85α) subunits of PI3K whereas **B** SA up-regulates eNOS mRNA expression. HAEC cells were incubated with 100 µM PA (black bars), OA (white bars), LA (grey bars) and SA (dark grey bars) for 24 h. Data are presented as the fold change in mRNA expression (normalised for β-actin) relative to the BSA control (HAEC incubated with BSA equivalent to the amount present in the fatty acid-BSA complexes), which is arbitrarily set as 1. The real time RT-PCR was performed in duplicate, and values represent mean ± SEM for three independent experiments. Insulin receptor expression *P = 0.03 relative to LA and **P < 0.02, relative to PA and SA. P110β gene expression ^§^*P* = 0.003 relative to LA and ^§§^*P* ≤ 0.004 relative to LA and SA. P85α expression ^¶^*P* ≤ 0.032 compared with LA and SA. eNOS gene expression ^#^*P* < 0.03 relative to PA, OA and LA

Fatty acid type x time interactions were observed for the mRNA expression of the insulin receptor (*P* = 0.001), p110β (a catalytic subunit of PI3K, *P* = 0.025), p85α (a regulatory subunit of PI3K, *P* = 0.011) and eNOS (*P* = 0.001), with differences among the fatty acids only observed after the 24 h incubation (Fig. 1 and Table S1). Compared with PA and stearic acid (SA), OA downregulated the expression of the insulin receptor (*P* ≤ 0.017) whereas a greater expression was observed with SA than LA (*P* = 0.030). Similarly, OA down-regulated the expression of p110β compared with LA and SA (*P* ≤ 0.004), with a greater up-regulation with SA than LA (*P* = 0.003). OA also down-regulated the expression of p85α compared with PA and LA (*P* ≤ 0.032) (Fig. 1A). A greater up-regulation of the expression of eNOS was observed with SA relative to the other fatty acids tested (*P* < 0.03) (Fig. 1B).

**Fig. 2:**
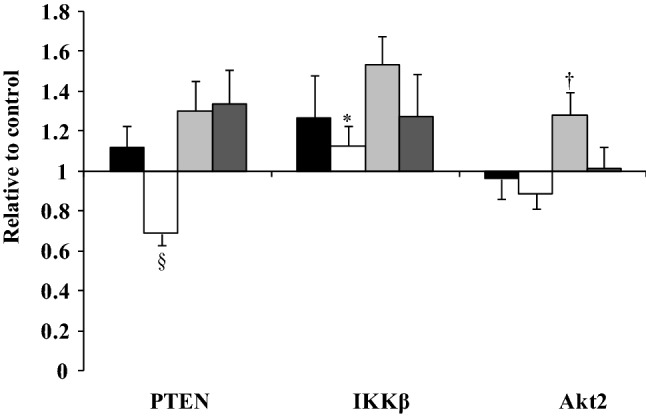
Differential effects of 100 µM PA (black bars), OA (white bars), LA (grey bars) and SA (dark grey bars) on the mRNA expression of PTEN, IKKβ and Akt2 (data for the 3 and 24 h incubations combined). Each bar represents the fold change in mRNA (normalised for β-actin) relative to the BSA control, which is arbitrarily set as 1. The real time RT-PCR was performed in duplicate, and values represent mean ± SEM for three independent experiments. ^§^*P* ≤ 0.05, relative to PTEN expression following PA, LA and SA; *P = 0.026, relative to IKKβ expression following LA; ^†^*P* < 0.04, relative to Akt2 expression following OA and SA

Significant effects of fatty acid type (data for the 3 and 24 h incubations combined) were observed on the mRNA expression of PTEN (*P* < 0.0001), IKKβ (*P* = 0.023), Akt2 (v-akt murine thymoma viral oncogene homologue 2) (*P* = 0.009) and VCAM-1 (*P* = 0.041). OA was shown to down-regulate the expression of PTEN compared with the PA, LA and SA (*P* ≤ 0.05). IKKβ gene expression was greater with LA than OA (*P* = 0.026), with a greater expression of Akt2 with LA compared with either OA (*P* = 0.015) or SA (*P* = 0.038)(Fig. 2). For VCAM-1, differences were not evident between PA, OA, SA and LA after post-hoc analysis.

**Fig. 3:**
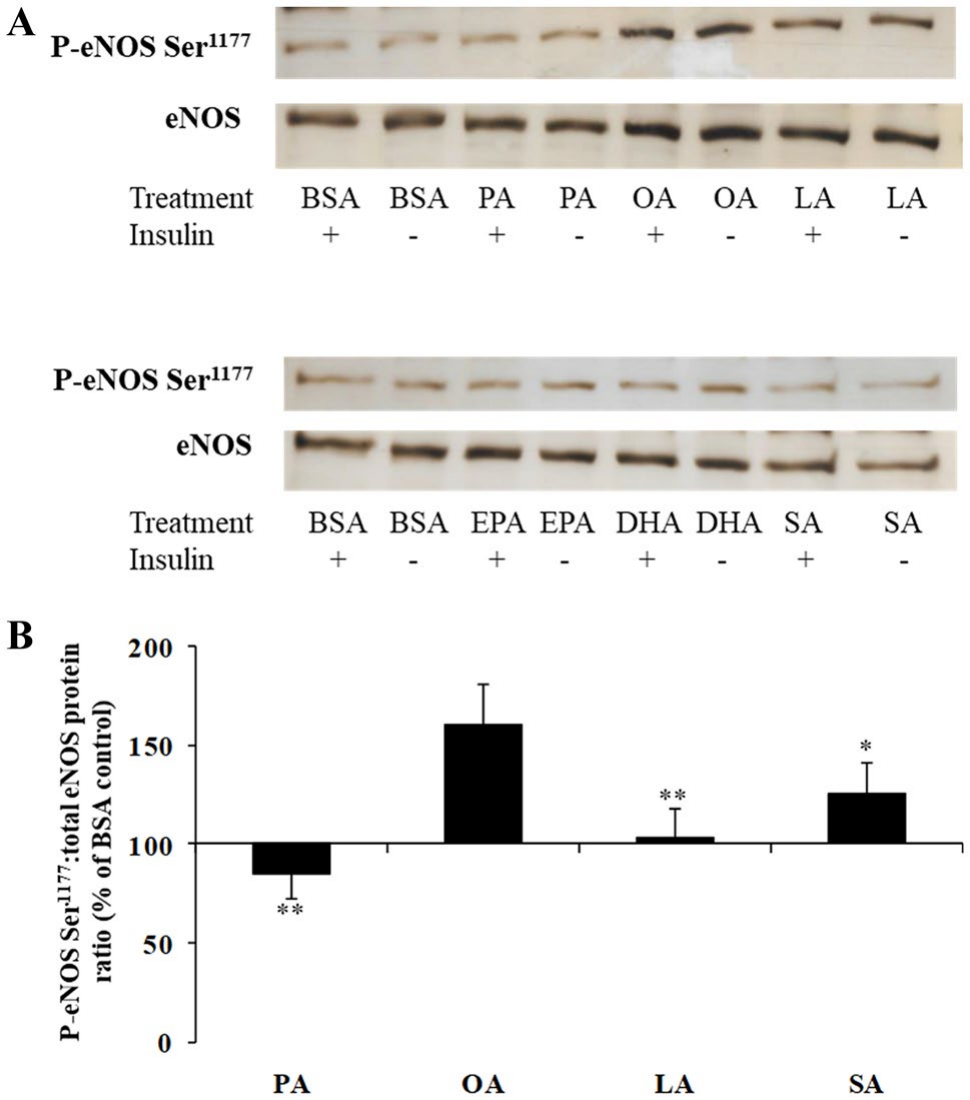
**A** A typical Western blot analysis of phosphorylated eNOS Ser^1177^ and total eNOS after incubation of HAEC cells with 100 µM PA, OA, LA and SA, and 10 µM EPA and DHA followed by stimulation with and without insulin (1 µM) for 15 min. **B** Greater eNOS phosphorylation at Ser^1177^ with OA. Each bar represents the phosphorylated:total eNOS protein ratio after incubation of HAEC with 100 µM of PA, OA, SA and LA relative to the BSA control, which is arbitrarily set at 100%. Data are pooled from several independent cell experiments (*n* = 10–12). **P ≤ 0.009 and *P = 0.047, relative to OA

In our cell signalling studies, there was a significant effect of fatty acid type on eNOS phosphorylation at Ser^1177^, calculated relative to the BSA control (*P* = 0.013) independent of whether the cells were stimulated with insulin or HBSS. Incubation of HAEC with 100 µM OA was associated with greater eNOS phosphorylation at Ser^1177^ than 100 µM PA, SA and LA (ratios after stimulation with HBSS and insulin combined; *P* ≤ 0.047) (Fig. 3). No significant effects were seen in the phosphorylation of Akt at Ser^472^ or p42/44 MAPK at Thr^202/204^ after the single fatty acid treatments, stimulated with HBSS or insulin (data not shown).

### Time-dependent effects of LC n-3 PUFA on vascular cell adhesion molecule-1 expression

**Fig. 4:**
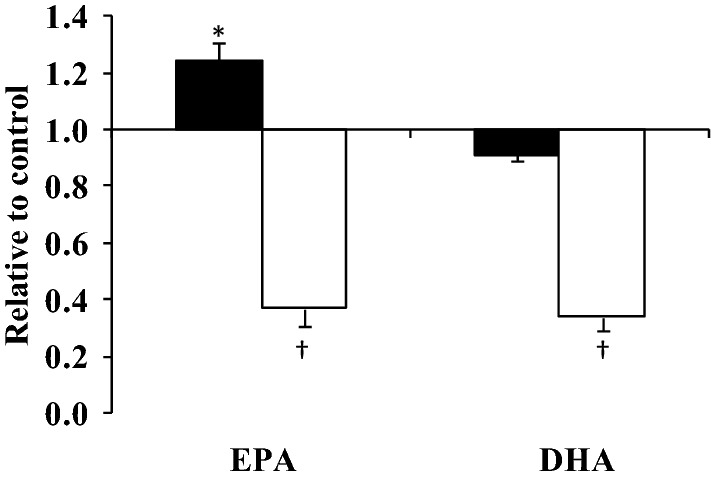
Time-dependent effects of LC n-3 PUFA on VCAM-1 mRNA expression. HAEC were incubated for 3 h (black bars) and 24 h (white bars) with 10 µM of EPA and DHA. Each bar represents the fold change in mRNA (normalised for β-actin) relative to the BSA control, which is arbitrarily set as 1. The real time PCR was performed in duplicate, and values represent mean ± SEM for three independent experiments. *P = 0.009, compared with 3 h mRNA expression with DHA; ^†^*P* ≤ 0.001, compared with the corresponding mRNA expression for the 3 h time point

Less marked effects were observed on the gene expression of proteins in the PI3K/Akt/eNOS pathway following incubation with 10 µM EPA and DHA. A significant effect of fatty acid type was observed on VCAM-1 expression only (*P* = 0.05), with a tendency for a fatty acid type x time interaction (*P* = 0.08). At 3 h, VCAM-1 expression was significantly greater following incubation with EPA than DHA (*P* = 0.009), with a marked down-regulation in expression observed with both fatty acids at 24 h (*P* ≤ 0.001) (Fig. 4).

### Both concentration and composition of the fatty acid mixtures influences expression of key proteins in the PI3K/Akt pathway

**Fig. 5:**
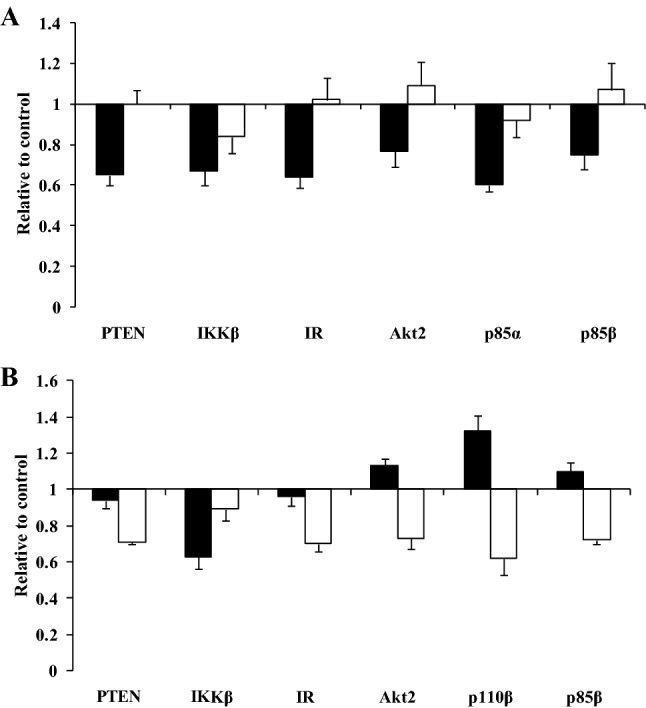
Significant effects of **A** concentration (400 µM (black bars) versus 800 µM (white bars)) and **B** composition [SFA (black bars) and SFA with LC n-3 PUFA (white bars)] of the fatty acid mixtures on the mRNA expression (normalised for β-actin) of key proteins in the PI3K/Akt signalling pathway. Each bar represents the fold change in mRNA relative to the BSA control, which is arbitrarily set as 1. The real time RT-PCR was performed in duplicate, and values represent mean ± SEM for three independent experiments. To determine the impact of fatty acid mixture concentration (**A**), gene expression data for the SFA and SFA with LC n-3 PUFA mixtures were combined whereas for fatty acid composition (**B**), data for the 400 µM and 800 µM incubations were combined

The two fatty acid mixtures, SFA and SFA with LC n-3 PUFA, were incubated at two concentrations, 400 µM and 800 µM, for a period of 3 h. Although there were no significant fatty acid mixture × concentration interactions, to explore the effects of NEFA concentration representative of normal (400 µM) and insulin resistant (800 µM) states, the fold change in gene expression for the SFA and SFA with LC n-3 PUFA fatty acid mixtures were combined. Significant effects of concentration were observed on the mRNA expression of PTEN (*P* = 0.005), IKKβ (*P* = 0.035), insulin receptor (*P* = 0.012), Akt2 (*P* = 0.008), p85α (*P* = 0.021) and p85β (*P* = 0.033), with each gene showing a greater down-regulation of mRNA expression with the 400 µM than 800 µM concentration (Fig. 5A).

To determine the overall impact of the fatty acid composition of the mixtures (SFA and SFA with LC n-3 PUFA), the fold change in gene expression for the 400 µM and 800 µM incubations were combined. Significant effects were only evident for the mRNA expression of PTEN (*P* = 0.009), IKKβ (*P* = 0.043), insulin receptor (*P* = 0.012), Akt2 (*P* = 0.006), p110β (*P* = 0.006) and p85β (*P* = 0.002) between the mixtures. In general, the SFA with LC n-3 PUFA mixture led to a down-regulation of the gene expression relative to the SFA mixture apart from IKKβ where a greater down-regulation of expression was observed with the SFA mixture (Fig. 5B).

The cell signalling studies revealed no significant effect of the concentration or type of fatty acid mixture on phosphorylation of eNOS at Ser^1177^, Akt at Ser^473^ or p44/42 MAPK at Thr^202^/Thr^204^ (data not shown).

## Discussion

Raised NEFA enriched in SFA are proposed to impair vascular function by attenuating insulin signalling via vasodilator pathways (PI3K/Akt/eNOS) and enhancing signalling via pro-inflammatory pathways (MAPK). However, very little is known about the effects of different fatty acid classes on these pathways in endothelial cells. In this in vitro study, we have reported that single fatty acids have differential effects on the expression of genes in the PI3K/Akt/eNOS pathway. Secondly, both the concentration and composition of the fatty acid mixtures, representative of circulating NEFA profiles from our previous human study, had significant influences on gene expression.

### Individual fatty acids

Insulin mediates its effects by binding to insulin receptors and triggering the interaction between IRS-1 and Class 1A PI3K, heterodimeric molecules consisting of a catalytic subunit (p110) and regulatory subunit (p85). This leads to the generation of phosphatidylinositol (3,4,5)-triphosphate (PIP_3_) which acts as a second messenger activating Akt/protein kinase B, with Akt2 proposed to be the key downstream intermediate linked to insulin action in vascular endothelial cells [22, 23]. In general, OA down-regulated the expression of the insulin receptor, p110β and p85α compared with PA, SA and LA, whereas SA increased expression of the insulin receptor and p110β relative to LA. As far as the authors are aware, this study is the first to determine the effects of fatty acids on the expression of both the catalytic (p110α and β) and regulatory subunits (p85α and β) of the Class 1A PI3K, which are proposed to have opposing effects on insulin signalling [24]. Studies in knock-out mice suggest that the regulatory subunits may have negative effects on insulin action [25], with increased expression of p85α also observed in mouse models of gestational diabetes [26] and in obese humans [8, 27]. Conversely, reductions in p85α (and to a lesser extent p85β) have been shown to enhance insulin sensitivity [24, 28] and lead to a decrease in the catalytic subunits p110α and p110β in the insulin sensitive tissues of these knock-out animals [25]. Interestingly, our observations for OA of a decrease in p85α and p110β expression suggest a co-ordinated down-regulation of these two subunits by OA and may indicate a direct effect of this fatty acid on PI3K. In agreement, opposing effects of 100 µM OA and PA on the mRNA expression of p85α have been reported in visceral adipose tissue biopsies from morbidly obese individuals, albeit the reduction in p110β expression was only evident at 25 µM but not 100 µM OA in this cell type [8]. Differences in the responsiveness of p110β to the actions of OA between studies may reflect the cell types used (endothelial cells versus visceral adipose tissue) and the phenotype of the adipose tissue donors (obese participants). Evidence from cancer cell lines have indicated that OA may activate PI3K by binding to the G protein-coupled receptors (GPCR, such as GPCR40 and 120), leading to transactivation of epidermal growth factor receptors via Src, activating a wide variety of pathways including PI3K/Akt [29, 30]. Although PI3K have been shown to be activated by tyrosine kinases, such as the insulin receptor, and by GPCR, the latter pathway has been shown to be inactivated by SFA [31]. Therefore, it is possible that OA may be influencing the expression of the PI3K subunits by binding to certain GPCR.

In addition to its well established effects on PI3K activation, it has been proposed that the regulatory subunit p85α forms part of a complex feedback regulatory system which modulates insulin signalling by activating PTEN [32, 33], one of the lipid phosphatases reported to antagonise the PI3K/Akt pathway by dephosphorylating PIP_3_ [34]. In vitro studies have previously reported fatty acid effects on PTEN expression in a number of different cell types [5, 35–37]. Up-regulation of expression was observed in endothelial cells after incubation with 200–800 µM PA [5] and in a breast cancer cell line with 50 ng/ml (approximately 15 µM) of EPA and DHA [35], whereas 50 µM [36] and 500 µM [37] of OA down-regulated PTEN in HepG2 cells. In agreement with this literature, we also observed a down-regulation of PTEN in HAEC with OA at 100 µM, with an up-regulation of mRNA expression with both SFA (PA and SA) and LA. The co-ordinated down-regulation of p85α, p110β and PTEN with OA suggests a promotion of signalling via PI3K/Akt, and this finding is supported by the greater eNOS phosphorylation at Ser^1177^ found with OA relative to PA, LA and SA. Improvements in insulin sensitivity with MUFA-rich diets have previously been observed, but findings have been inconsistent in both healthy and insulin resistant adults [38–40], with evidence to suggest that MUFA-rich diets or test meals may also lead to impaired vascular reactivity. However, insulin stimulation of the cells after fatty acid treatment did not reveal any further differences in phosphorylation at eNOS at Ser^1177^ or Akt at Ser^473^, but this may reflect the dose of insulin (1 µM) used in the cell experiments, which is known to stimulate insulin-like growth factor 1 receptor in addition to the insulin receptor [41].

In the present study, differences in the expression of the insulin receptor were also observed after 24 h, with a lower expression with OA relative to PA and SA. Our findings are in contrast with previous observations which have shown a decrease in expression and post-receptor signalling with a range of PA concentrations (250–750 µM) in different cell types [42–44]. However, the effects of unsaturated fatty acids were not included in these previous studies. Our observations of an up-regulation in expression after 24 h with SFA, relative to OA, may be explained by the type of cells used in this in vitro study (hepatocytes are a more anabolic tissue than endothelial cells), or the lower concentration of fatty acids treatments (100 µM), and/or the level of signalling via the PI3K/Akt pathway. The marked effect of OA on the PI3K/Akt/eNOS pathway is intriguing and further work is warranted to determine the primary site of regulation of this pathway by OA. Potential candidates could include the insulin receptor, PI3K regulatory subunits, PTEN or certain GPCR.

It has been demonstrated that IKKβ, the serine kinase which regulates the activation of NFκB, also uses IRS-1 as a substrate for serine phosphorylation, with activation of IKKβ providing a key pathway for the attenuation of insulin signalling via PI3K/Akt [13]. Although a greater IKKβ mRNA expression was observed with LA than OA, the induction of gene expression for the cell adhesion molecule VCAM-1 by NFκB was not evident after incubation with PA, SA, LA and OA, with differential effects on expression only observed with the LC n-3 PUFA. Acute exposure of the cells caused an up-regulation of VCAM-1 mRNA expression with EPA compared with DHA, whereas the chronic 24 h exposure down-regulated expression to the same extent following both fatty acids. These time dependent effects of LC n-3 PUFA on VCAM-1 expression have previously been observed in our group using human umbilical vein endothelial cells [11] highlighting the importance of length of fatty acid exposure on gene expression and cell signalling, since changes in membrane composition may occur during the longer incubations. Increased eNOS levels have been associated with reduced inflammation via the effects of the vasodilator nitric oxide on VCAM-1 expression [45]. Compared with the control, incubation with SA produced a 4.5-fold greater eNOS mRNA expression compared with PA (2.5 fold) and unsaturated fatty acids (1.1 fold) which was mirrored in the VCAM-1 expression, although the differences between fatty acids were not significant. As suggested previously, a negative feedback may exist in which cells up-regulate eNOS expression to reverse the inflammatory effects mediated by the fatty acid exposures [11].

### Fatty acid mixtures—effects of concentration and composition

Elevated NEFA concentrations are characteristic of conditions such as obesity and type II diabetes. Incubation with the fatty acid mixtures at 400 and 800 µM revealed differential effects on the expression of the insulin receptor, regulatory subunits of PI3K (p85α and β), IKKβ and Akt2, with a consistent down-regulation of expression with 400 µM compared with 800 µM. A greater down-regulation of p85α, p85β, PTEN and IKKβ is generally consistent with the possibility that the 400 µM concentration (typical fasting level for humans) is likely to be associated with greater insulin sensitivity than with the 800 µM concentration (levels observed in diabetes or after a very high fat meal). However, the reduction in Akt2 expression conflicts with this interpretation since Akt knock-out animals exhibit a diabetic phenotype consistent with insulin resistance [46]. It is possible that the reduction in expression with 400 µM may reflect an adaptive response to the down-regulation of the PI3K subunits and PTEN, which are upstream of Akt, or alternatively a compensatory effect associated with changes in other Akt isoforms present in the endothelial cells.

We have previously shown experimental elevation of NEFA enriched in SFA with LC n-3 PUFA to reverse the detrimental effects of SFA on flow mediated dilatation in healthy adults [3]. Incubation with a fatty acid mixture representative of the plasma NEFA fatty acid composition after the SFA with LC n-3 PUFA test drinks led to a down-regulation of the insulin receptor, p110β, p85β, PTEN and Akt compared with the SFA mixture, with a greater down-regulation of IKKβ with the SFA mixture. The downregulation of PI3K subunits and PTEN suggests greater signalling via the PI3K/Akt pathway with the SFA and LC n-3 PUFA mixture which may have contributed to the improved vascular function. However, in our cell signalling studies, differences were not observed in the phosphorylation status of eNOS at Ser^1177^ or Akt at Ser^473^ between the fatty acid mixtures. Therefore, it is possible that other pathways or mechanisms may have played a more significant role in mediating the beneficial effects of LC n-3 PUFA on vascular function in our human study, such as direct effects of the fatty acids on the smooth muscle cell layer and bioavailability of nitric oxide [47].

A strength of this in vitro study is the use of concentrations reflective of circulating individual fatty acids and the comparison with fatty acid mixtures representative of postprandial plasma NEFA after meal ingestion. Our findings highlight the differences in the magnitude of the effects of the fatty acids on mRNA gene expression when they are incubated with the cells alone versus in combination with other dietary fatty acids. We have previously observed a similar phenomenon on the expression of genes involved in endothelial activation following incubation with single dairy fatty acids and mixtures representative of low and high dairy intakes [48]. This highlights the need for careful interpretation of findings from in vitro and ex-vivo studies using single fatty acids. However, this study also has some limitations. We used a primary endothelial cell line to determine the mechanisms of action of fatty acids on the expression of genes involved in the PI3K/Akt pathway and eNOS phosphorylation which does not represent the in vivo situation in which cells lining the blood vessel wall are in close contact with the underlying smooth muscle layer. Our study also focussed specifically on key genes involved in the PI3K/Akt pathway and so we cannot discount that other genes or proteins involved in this pathway may have been mediating the effects observed. Furthermore, cell medium nitric oxide concentrations and HAEC membrane FA compositions were not measured following the acute and chronic incubations in which to relate to the eNOS phosphorylation data and gene expression data. Studies are needed which examine the insulin signalling pathways in the endothelium in greater detail.

## Conclusion

We report for the first time the effects of OA on the mRNA expression of the regulatory (p85α) and catalytic (p110β) subunits of PI3K in endothelial cells, in addition to confirming previous observations of this fatty acid on p85α [8] and PTEN expression [36]. These findings may, in the case of some of the reported effects, appear counter intuitive or contrary to what may have been predicted. However, together with the cell signalling studies, our data suggests effects of OA on the expression of the PI3K/Akt/eNOS pathway are consistent with improved insulin sensitivity observed in knock-out animal studies, where down-regulation of the PI3K regulatory subunits appear to be inversely correlated with insulin sensitivity. Furthermore, our findings also contribute to a better understanding of how both the concentration and composition of fatty acid mixtures may influence endothelial function by modulation of the PI3K/Akt pathway. In view of the increasing prevalence of the metabolic syndrome and type II diabetes in the population, further work is required to determine the underlying molecular basis of insulin resistance in the endothelium and the modulatory effects of dietary lipids.

## Supplementary Information

Below is the link to the electronic supplementary material.

Supplementary file1 (DOCX 14 KB)

## Data Availability

All data generated or analysed during this study are included in this published article and its associated Supplementary Materials files. The data that support the findings of this study are available from the corresponding author upon reasonable request.

## Abbreviations

Akt2: V-akt murine thymoma viral oncogene homologue 2
BSA: Bovine serum albumin
DHA: Docosahexaenoic acid
eNOS: Endothelial nitric oxide synthase
EPA: Eicosapentaenoic acid
FBS: Foetal bovine serum
GCPR: G coupled protein receptor
HAEC: Human aortic endothelial cells
IKKβ: Inhibitor-κβ kinase-β
IR: Insulin receptor
IRS-1: Insulin receptor substrate-1
LA: Linoleic acid
LC: Long chain
LVM: Large vessel medium
MAPK: Mitogen-activated protein kinase
MUFA: Monounsaturated fatty acids
NEFA: Non-esterified fatty acids
NFκB: Nuclear factor kappa-B
OA: Oleic acid
PA: Palmitic acid
PI3K: Phosphoinositide 3-kinase
PTEN: Phosphatase and tensin homolog
PUFA: Polyunsaturated fatty acids
SA: Stearic acid
SFA: Saturated fatty acids
VCAM: Vascular cell adhesion molecule
