# Supplementary material for: Differential effects of single fatty acids and fatty acid mixtures on the phosphoinositide 3-kinase/Akt/eNOS pathway in endothelial cells
Source: Eur J Nutr. 2022 Feb 14;61(5):2463–73. doi: 10.1007/s00394-022-02821-2 (PMC9279250; doi:10.1007/s00394-022-02821-2)
Supplement: Supplementary file 1 — Supplementary file1 (DOCX 14 KB) [file 394_2022_2821_MOESM1_ESM.docx]

**Supplementary Table 1**: Acute (3 h) and chronic (24 h) of effects 100 µM PA, OA, LA and SA on mRNA expression in human aortic endothelial cells.

| **Gene** | **Fatty acid** | | | | **ANOVA** | |
| --- | --- | --- | --- | --- | --- | --- |
|  | PA | OA | LA | SA | fatty acid*time (*P*=) | fatty acid (*P*=) |
| Akt2  3 h  24 h | 0.84 ± 0.16  1.08 ± 0.08 | 0.77 ± 0.12  1.00 ± 0.05 | 1.08 ± 0.11  1.49 ± 0.09 | 0.81 ± 0.09  1.21 ± 0.10 | 0.699 | 0.005 |
| IKKβ  3 h  24 h | 0.82 ± 0.06  1.72 ± 0.11 | 0.91 ± 0.03  1.35 ± 0.04 | 1.34 ± 0.11  1.73 ± 0.21 | 0.82 ± 0.10  1.73 ± 0.05 | 0.062 | 0.023 |
| IR  3 h  24 h | 0.95 ± 0.02  1.36 ± 0.13 | 1.01 ± 0.12  0.84 ± 0.09 | 0.78 ± 0.12  1.21 ± 0.08 | 0.62 ± 0.14  1.65 ± 0.05 | 0.001 | 0.099 |
| p85α  3 h  24 h | 0.89 ± 0.21  1.32 ± 0.13 | 0.87 ± 0.08  0.67 ± 0.02 | 0.77 ± 0.09  1.64 ± 0.25 | 0.87 ± 0.15  1.66 ± 0.26 | 0.002 | 0.011 |
| p85β  3 h  24 h | 0.85 ± 0.11  1.47 ± 0.22 | 0.89 ± 0.11  1.29 ± 0.07 | 0.88 ± 0.07  1.57 ± 0.22 | 0.63 ± 0.14  1.23 ± 0.08 | 0.814 | 0.301 |
| p110α  3 h  24 h | 0.89 ± 0.11  1.46 ± 0.32 | 1.16 ± 0.05  1.07 ± 0.1 | 0.97 ± 0.07  1.35 ± 0.36 | 0.80 ± 0.12  1.31 ± 0.21 | 0.437 | 0.945 |
| p110β  3 h  24 h | 0.74 ± 0.16  1.20 ± 0.24 | 0.57 ± 0.08  0.46 ± 0.05 | 0.70 ± 0.16  1.13 ± 0.01 | 0.64 ± 0.02  1.38 ± 0.02 | 0.025 | 0.005 |
| PTEN  3 h  24 h | 0.92 ± 0.08  1.32 ± 0.09 | 0.56 ± 0.02  0.81 ± 0.05 | 1.06 ± 0.04  1.54 ± 0.22 | 1.01 ± 0.07  1.66 ± 0.19 | 0.233 | <0.001 |
| eNOS  3 h  24 h | 0.90 ± 0.15  2.46 ± 0.70 | 0.90 ± 0.08  1.04 ± 0.20 | 0.58 ± 0.09  1.05 ± 0.13 | 0.94 ± 0.18  4.25 ± 0.94 | 0.001 | <0.001 |
| VCAM-1  3 h  24 h | 0.65 ± 0.20  1.57 ± 0.19 | 0.75 ± 0.07  0.95 ± 0.17 | 0.46 ± 0.03  1.09 ± 0.19 | 0.70 ± 0.12  2.84 ± 0.89 | 0.057 | 0.041 |

The fold change in mRNA is calculated relative to the control (HAEC incubated with BSA equivalent to the amount present in the fatty acid-BSA complexes), which is arbitrarily set as 1. The real time RT-PCR was performed in duplicate, and values represent mean ± SEM for three independent experiments for each fatty acid.

Abbreviations: Akt2, v-akt murine thymoma viral oncogene homologue 2; eNOS, endothelial nitric oxide synthase; IKKβ, inhibitor-κβ kinase-β; IR, insulin receptor; PI3K regulatory (p85α and p85β) and catalytic (p110α and p110β) subunits; PTEN, phosphatase and tensin homolog; VCAM, vascular cell adhesion molecule.
